# Therapeutic Effect of *Tetrapanax papyriferus* and Hederagenin on Chronic Neuropathic Pain of Chronic Constriction Injury of Sciatic Nerve Rats Based on KEGG Pathway Prediction and Experimental Verification

**DOI:** 10.1155/2020/2545806

**Published:** 2020-06-13

**Authors:** Di Zhang, Jianxin Sun, Bing Yang, Shengsuo Ma, Chunlan Zhang, Guoping Zhao

**Affiliations:** College of Traditional Chinese Medicine, Jinan University, Guangzhou 510630, China

## Abstract

**Background:**

Hederagenin is one of the main components of *Tetrapanax papyriferus*, and *Tetrapanax papyriferus* is one of the ingredients of Danggui Sini decoction. To explore whether *Tetrapanax papyriferus* and hederagenin can alleviate mechanical pain, thermal hyperalgesia, and cold pain at the same time, we comprehensively investigated the effects of two drugs on the levels of p38 MAPK phosphorylation, TRP proteins, and IL1*β*, IL6, and TNF-*α* in serum.

**Methods:**

Firstly, we obtained pain-related targets and performed KEGG pathway enrichment on these targets. Then, 42 SD rats were separated randomly into six groups: sham operation group, CCI group, pregabalin group, mecobalamin group, *Tetrapanax papyriferus* group, and hederagenin group. All drugs were given orally. Rats in the sham operation group and CCI group were gavaged with saline. Rats in the pregabalin group were given pregabalin, while rats in the mecobalamin group were given mecobalamin. Rats in the *Tetrapanax papyriferus* group were given *Tetrapanax papyriferus*, while rats in the hederagenin group were given hederagenin. Besides, we conducted behavioral tests including acetone test, hot plate experiment, and von Frey filaments, and then dorsal root ganglion neurons were taken out on the 21st day after operation. Then, western blot, ELISA, and hematoxylin-eosin staining were conducted.

**Results:**

Rats in the CCI group were more sensitive to hyperalgesia and allodynia to mechanical and thermal stimuli, as well as cold pain. All four drugs could relieve these pains. Pregabalin, mecobalamin, and *Tetrapanax papyriferus* can reduce the levels of IL1*β*, IL6, and TNF-*α* in serum compared to those of the CCI group. The expression of TRPM8, TRPA1, TRPV1, TRPV4, and phosphorylated p38 MAPK in DRG increased evidently on the 21st day after the operation in the CCI group. All four drugs could reduce the expressions of TRPM8, TRPA1, TRPV1, TRPV4, and phosphorylated p38 MAPK in dorsal root ganglion compared to those of the CCI group.

**Conclusion:**

*Tetrapanax papyriferus* and hederagenin relieved sciatica by reducing inflammation levels, inhibiting p38 MAPK phosphorylation, and decreasing the levels of dorsal root ganglion proteins.

## 1. Introduction

Chronic neuropathic pain, triggered by peripheral nerve injury, is defined as unpleasant sensations of burning and tingling with increased sensitivity towards pain. This pain arises as a direct response to a lesion or disease affecting the somatosensory system. Given the pain relief, mainstream analgesics are not sufficiently successful in achieving selective palliation of neuropathic pain. Beside the pharmacological therapies, such as gabapentin and pregabalin, people have tried to develop nonpharmacological methods for treating pain including physical therapy, psychotherapy, traditional Chinese medicine, and nerve stimulation therapy [1]. Among them, traditional Chinese medicine has attracted great attention. Recent research showed that Danggui Sini decoction, a traditional Chinese medicine formula, has been widely used as a remedy for neuropathic pain and other diseases [2].

A gene family involved in pain sensory function is the transient receptor potential (TRP) channel superfamily [3]. TRPA1 is a universal chemo-irritant receptor involved in neuropathic cold pain. TRPM8 expression is restricted to a subset of small-diameter sensory neurons in the trigeminal and dorsal root ganglia [4]. Besides, TRPM8 has been mechanistically linked to cold hypersensitivity [5]. TRPV1 is preferentially expressed in sensory neurons of the peripheral nervous system, specifically in laminae I and II of the dorsal horn of the spinal cord, where it modulates the synaptic transmission of nociceptive signals from the periphery [6]. TRPV4 in sensory neurons can be sensitized by proinflammatory mediators, such as prostaglandin E2, an integrator of proteolytic signaling in inflammation [7, 8]. TRPV4 has been implicated in nerve pain in several preclinical rodent pain models, such as paclitaxel-induced neural injury leading to painful peripheral neuropathy and chronic mechanical compression-injury (CCI) of the DRG [9].

Hederagenin is one of the main components of *Tetrapanax papyriferus*, and *Tetrapanax papyriferus* is one of the ingredients of Danggui Sini decoction. There is a series of related studies about these two drugs. Namki found that triterpenes from *Tetrapanax papyriferus* reduced the LPS-induced expression of proinflammatory cytokines, such as TNF-*α* and IL-6, and could be a potential natural resource of NO inhibitors used in the treatment of neurodegenerative disorders [10]. Kim showed increased expression of the apoptosis-associated protein, Bcl-2, and decreased expression of Bax and p53 after treatment with hederagenin, and hederagenin treatment attenuated ethanol-induced increases in activated p38 MAPK and increased the levels of phosphorylated AKT and ERK [11]. Our previous research found that Danggui Sini decoction can not only suppress the cold allodynia of neuropathic pain of the CCI rats but also alleviate its mechanical allodynia and thermal hyperalgesia [12]. To discover the analgesic ingredients of Danggui Sini decoction, we suppose that *Tetrapanax papyriferus,* the ingredient of Danggui Sini decoction, and hederagenin might attenuate pain allergy of CCI rats. Therefore, we hypothesized that *Tetrapanax papyriferus* and hederagenin can relieve sciatica by reducing inflammation levels, inhibiting p38 MAPK phosphorylation, and decreasing the levels of TRP proteins.

## 2. Materials and Methods

### 2.1. Chronic Neuropathic Pain-Related KEGG Pathways Prediction

By inserting the keywords “chronic neuropathic pain” into the GeneCards database (https://www.genecards.org), we searched for the reported chronic neuropathic pain-related genes and removed the false positive genes. Then, the results were imported into the WebGestalt database (http://www.webgestalt.org/option.php), and KEGG pathway analysis was carried out.

### 2.2. Subjects

Male Sprague Dawley rats, weighing 200–230 g, aged 7-8 weeks, were purchased from the Experimental Center of Beijing Huafu Kang Co. Ltd. (license no. SCXK 2014-0004:11401300091361). Four rats were nourished in individual cages at the room, with controlled temperature (20–22°C) and humidity (40–60%), and were fostered on an alternative 12 h light/dark cycle in the Animal Experimental Center of Medical College of Jinan University SPF animal housing management (ethical number: 20181008-04). All rats were allowed to accommodate to laboratory conditions for 10 days before starting the experiments and were provided with food and water freely. All animal studies conformed to the Regulations of Experimental Animal Administration promulgated by the State Committee of Science and Technology of China on November 14, 1988.

### 2.3. Drugs

Hederagenin (MB5701) was purchased from Guangzhou Jiayan Co. Ltd. *Tetrapanax papyriferus* was from Guangzhou Hexiang Pharmaceutical Co. Ltd. The PBS buffer,Tween 20, TBS, and neutral balsam was acquired from Saiguo Biological Technology Co. Ltd. (China). PageRuler Prestained Protein Ladder and Marker were obtained from Thermo (Waltham, US). The PVDF membrane was obtained from Millipore (Billerica, US). And BCA protein content test kit (AAPR161-A30) was obtained from Guangzhou Junji Biotechnology Co. Ltd. (China), and RIPA was purchased from Guangzhou Dingguo Biological Co. Ltd. (China). Polyclonal rat anti-rabbit TRPV1, p38 MAPK, phospho-p38 MAPK, TRPV4, TRPA1, TRPM8, and GAPDH were obtained from Guangzhou Juyan Biological Co. Ltd. (China). Following a sequence, these are the antibody numbers: b6166, 8690S, 4511S, NB110-74960, NB110-40763, NBP1-97311, and 5174S. And all these dilutions are 1 : 1000. Primary antidiluent, secondary antibody dilution, WB transfer solution, and WB electrophoresis solution were purchased from Beyotime Biotechnology (China). Phosphatase inhibitor cocktail 1 was from Guangzhou Jiayan Biological Technology Co. Ltd. (China). IL6 ELISA kit (ml102828), IL1*β* ELISA kit (ml003057), and TNF-*α* ELISA kit (ml002859) were purchased from Guangzhou Speed Research Biological Co. Ltd.

### 2.4. The Neuropathic Pain Model [13] (CCI)

Rats (*n* = 42) were anaesthetized with pentobarbital sodium (3%, 40 mg/kg, i.p., reinforced if necessary). The rats were then positioned in a stereotaxic frame. The sciatic nerve was exposed at the level of the middle of the thigh by blunt dissection through biceps femoris [14]. The bifurcated anterior nerve part of the sciatic nerve was tied (using 4.0 sutures) under a dissecting microscope and performed 4 times at intervals of about 1 mm, and a little twitch in the operated hindlimb was obtained, while rats (*n* = 7) in the sham group were subjected to all of these procedures but were not subjected to CCI. Then, gentamicin (2 ml, 10 mg/ml, i.m.) was given after the suture of the wound. After the surgery, sustained tactile and thermal hypersensitivity in the injured hind paw developed in each animal.

### 2.5. Drug Dosages and Method of Administration

42 SD rats were separated randomly into six groups: the sham operation group (SG), CCI group (CG), pregabalin group (PG), mecobalamin group (MG), *Tetrapanax papyriferu*s group (TG), and hederagenin group (HG). All rats received oral medication for 21 days. Rats in the sham operation group and CCI group were gavaged with saline (0.9% 6 mL/kg·d·bid). Rats in pregabalin group were given the pregabalin (10 mg/kg·d·bid), while rats in mecobalamin group were given mecobalamin (15 *μ*g/kg·d·bid). Rats in *Tetrapanax papyriferus* group were given *Tetrapanax papyriferus* (6.1 g/kg·d·bid), while rats in hederagenin group were given hederagenin (10 mg/kg·d·bid).

### 2.6. Behavioral Tests

Cold hyperalgesia was estimated by application of acetone. 100 *μ*l of acetone was projected via a 100 *μ*l pipette onto the plantar surface of each hind paw [15]. Acetone was propelled from below via air burst by expressing the pipette, thereby avoiding mechanical stimulation of the paw with the pipette. Total time of lifting or clutching each hind paw was recorded with a maximum cutoff time of 120 s. Cold stimulation was repeated 3 times at an interval of 10 min on each paw, and the mean calculated. Mechanical allodynia (50% MWT) was assessed withvon Frey filaments [15]. The plantar surface of each hind paw was stimulated ten times with each filament (2.0–26.0 g), starting with the 2 g filament and increasing until the rats responded by clutching and/or lifting the paw off the surface of the test apparatus. The mid-plantar ipsilateral and contralateral hind paw areas were tested. No significantly different contralateral hind paw reactions were observed between the CCI and naive rats. Paw lifting was coded as a positive response. Once a positive response was detected, sequentially lower weight filaments were used to estimate the sensory threshold for each paw. Then, the pain threshold should be calculated (50%*g* threshold = 10^[*Xf*+*kδ*]^/10000). Heat hyperalgesia (PWL) was evaluated by hot plate experiment. The lateral plantar surface was exposed to the heated plate (50°C). The initial withdrawal latency and duration were recorded. The heat stimulation was replicated 3 times at an interval of 10 min on each paw, and the mean calculated.

### 2.7. Hematoxylin-Eosin (HE) Stain

Ligation of the sciatic nerve was stored in 4% paraformaldehyde for 24 h, then embedded in paraffin, and sectioned in accordance with routine procedures [16]. After being deparaffinized with xylene, sections were cultured in ethanol (100%, 90%, 80%, and 70%, respectively) and then rinsed in PBS for 5 min. Then, a pathologist was assigned to test the severity of tissue damage by evaluating the inflammatory injury and neurochemical change.

### 2.8. ELISA Test

We got blood (8 ml) from the abdominal aorta of rats on the 21st day after the operation. The blood collection tube was centrifuged at 10,000 rpm for 20 minutes at 4°C. Then we obtained the supernatant (800 ul). The supernatant was taken to detect expressions of IL1*β*, IL6, and TNF-*α* by ELISA kits. And the samples being tested were taken out from the refrigerator 20–30 min before the beginning of the experiment to make their temperature reach room temperature. The solution was gently shaken at room temperature. The optical density value was analyzed by using a microplate reader, and the detection method was performed in strict accordance with the instruction of the kit [17].

### 2.9. Western Blots

To further explore the mechanisms, we examined the effects of these drugs on the numerous proteins. The expression changes of TRPA1, TRPM8, TRPV1, TRPV4, p38 MAPK, and p-p38 MAPK on DRGs were checked. DRGs (L4-L6), corresponding to the afferent pathway from the hind paw, were carefully removed and processed as previously described [18]. The DRGs were homogenized in RIPA lysis buffer, and the homogenates were centrifuged at 14,000°rpm at 4°C for 10 minutes. Total protein was extracted according to the protein extraction kit instructions. Protein concentration was determined using the BCA assay. After electrophoresis, the protein was taken to PVDF membranes and blocked with 5% skimmed milk powder for 1 h at room temperature. Membranes were incubated with primary antibody (diluted according to the manufacturer's instructions, just 1 : 1000) overnight at 4°C. The next day, membranes were incubated with 1 : 5000 dilution of horseradish peroxidase conjugated secondary antibody for 1 h at room temperature. After extensive washes with TBST three times, the PVDF membranes were detected by the gel automatic imaging system for exposure with the ECL kit. Western blotting had to be carried out using Image Lab software for grey value analysis.

### 2.10. Statistical Analyses

The number of animals used in the behavioral and biochemical studies was selected based on an earlier study on a similar field. All graphs and analyses were prepared using GraphPad Prism 8 and SPSS13.0 software. Repeated measure ANOVAs and SNK were performed to measure various data. The data and statistical analysis comply with the recommendations on experimental design and analysis in pharmacology [19].

### 2.11. Experimental Flow Chart

## 3. Results

### 3.1. KEGG Pathway Analysis of Pain-Rated Genes

**Figure 1:**
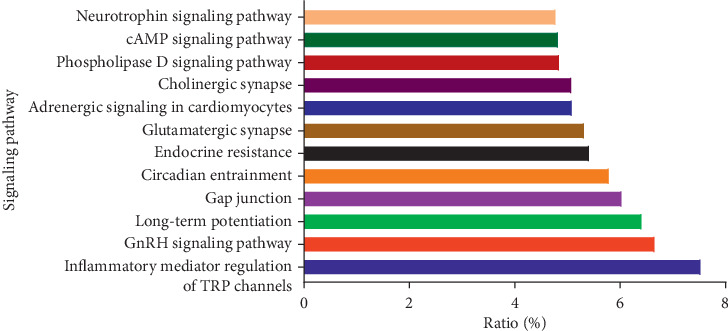
KEGG pathway analysis of chronic neuropathic pain-rated genes.

**Figure 2:**
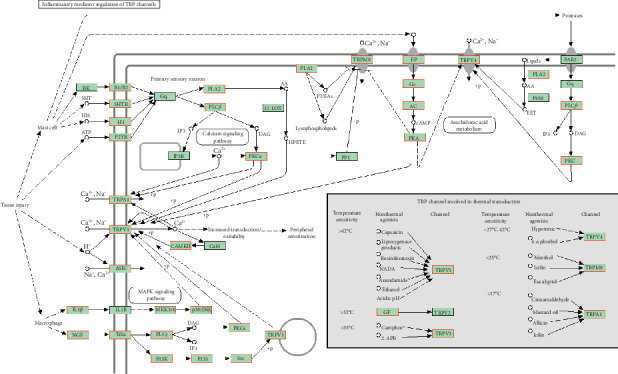
Inflammatory mediator regulation of TRP channels (^*∗*^red font part is pain related).

Through GeneCards, we obtained 788 chronic neuropathic pain-related genes. Then these were input into the WebGestalt. The first 12 KEGG pathways were selected and plotted in Figure 1, Table 1, and Figure 2 by the ratio. We chose the “inflammatory mediator regulation of TRP channels” pathway for the experimental verification because the rate ranked first.

### 3.2. Hot Plate Experiment (Thermal Withdrawal Latency)

**Figure 3:**
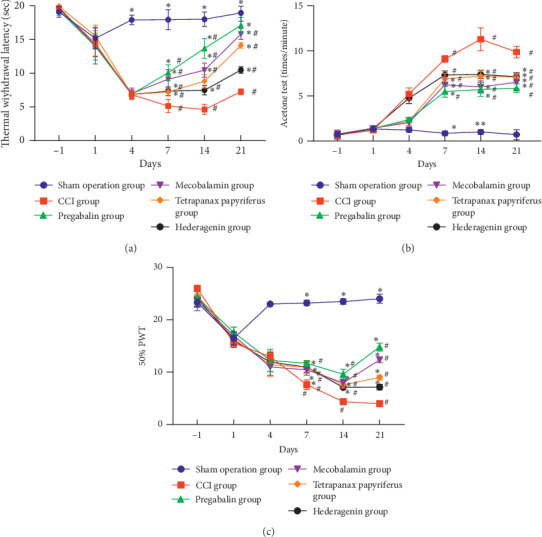
Behavioral testing of rats in each group. ^#^The contrast of the sham operation group and the other groups. ^*∗*^Comparison of the CCI group and the other groups.

This result (Figure 3(a)) indicated that sensitivity to heat pain appeared on the fourth day and reached a peak on the 14th day (4, 7, 14, 21 d, *p* < 0.05). Rats in the other groups were less sensitive to the thermal damage compared with the rats in the CCI group (7, 14, 21 d, *p* < 0.05). No Rats' thermal sensitivity returned to normal levels compared with sham-operated rats (7, 14, 21 d, *p* < 0.05), except for the performance of rats in the pregabalin group on the 21st day. The therapeutic effects of *Tetrapanax papyriferus* and hederagenin in the case of remission thermal hyperalgesia were as good as those of pregabalin and mecobalamin, and statistical analysis showed no difference.

### 3.3. Acetone Experiments

Results of the acetone experiment (Figure 3(b)) indicated that cold pain sensitivity in CCI group appeared on the fourth day, while it was enhancing during 7–10 days, reaching the highest point on the 14th day, and declining on the 21st day moderately. From the 7th day, compared with the rats in the CCI group, the rats in the other groups were less sensitive to cold stimulation (7, 14, 21 d, *p* < 0.05). Treated rats were more tolerant of cold pain than rats in the CCI group but did not reach normal levels as the rats in the sham group (7, 14, 21 d, *p* < 0.05). These drugs including *Tetrapanax papyriferus*, hederagenin, pregabalin, and mecobalamin had the same effect in treating cold pain, and statistics showed no difference.

### 3.4. Von Frey Experiment

The sequels (Figure 3(c)) manifested that the rats in the CCI group showed apparent mechanical pain sensitivity on the postoperative 4 days; however, the mechanical pain threshold on the 7th day was significantly reduced. Compared to the rats in the CCI group, the rats in other groups were less sensitive to mechanical pain (7, 14, 21 d, *p* < 0.05). Although various drugs could relieve the mechanical pain sensitivity, these could not make these rats return to normal (7, 14, 21 d, *p* < 0.05). The therapeutic effects of *Tetrapanax papyriferus* and hederagenin in relieving mechanical pain were as good as those of pregabalin and mecobalamin, and statistical analysis showed no difference.

Figure 3(a) indicated that rats in the other groups were less sensitive to thermal damage than the rats in the CCI model group (7, 14, 21 d, *p* < 0.05). And no rats' thermal sensitivity returned to normal levels compared with sham-operated rats (7, 14, 21 d, *p* < 0.05), except for the performance of rats in the pregabalin group on the 21st day. Figure 3(b) showed that the rats in the other groups were less sensitive to cold stimulation compared with the rats in the CCI model group (7, 14, 21 d, *p* < 0.05). Figure 3(c) manifested that the rats in the other groups were less sensitive to mechanical pain (7, 14, 21 d, *p* < 0.05) compared to the rats in the sham operation group. Although various drugs could relieve the mechanical pain sensitivity, these could not make these rats return to normal (7, 14, 21 d, *p* < 0.05).

### 3.5. Hematoxylin-Eosin (HE) Stain

**Figure 4:**
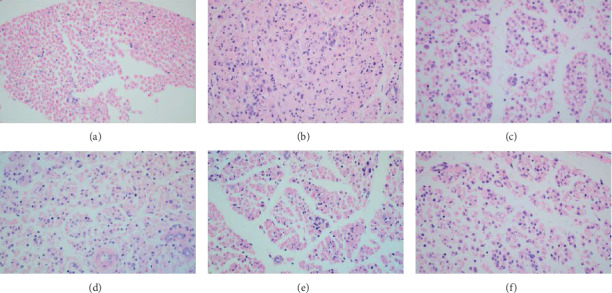
Hematoxylin-eosin staining of injured nerve. Rats in the sham operation group had intact nerve structure and no inflammatory cell infiltration (a). Proliferation of a large number of Schwann cells was also observed. Moreover, we observed that the normal structure of sciatic nerve was damaged (b). After treatments, we observed a decrease in inflammatory cells and Schwann cell proliferation, but the morphology of the sciatic nerve tissue structure did not return to normal (c–f). All pictures were taken under an optical 400x microscope. (a) Sham operation group. (b) CCI group. (c) Pregabalin group. (d) Mecobalamin group. (e) *Tetrapanax papyriferus* group. (f) Hederagenin group.

In the sham operation group on 21st day, the normal structure of the sciatic nerve can be observed (Figure 4(a)). We could hardly observe Schwann cells and inflammatory cell infiltration. On the 21st day after CCI operation, the inflammatory cell infiltration was observed in the CCI model group, and proliferation of a large number of Schwann cells was also observed. Moreover, we observed that the normal structure of sciatic nerve was damaged (Figure 4(b)). After treatments, we observed a decrease in inflammatory cells and Schwann cell proliferation, but the morphology of the sciatic nerve tissue structure did not return to normal (Figures 4(c)–4(f)). From the stained picture, we cannot compare the pros and cons of the four drugs. We can see these in Figure 4.

### 3.6. Detection of Inflammatory Factors in Serum

**Figure 5:**
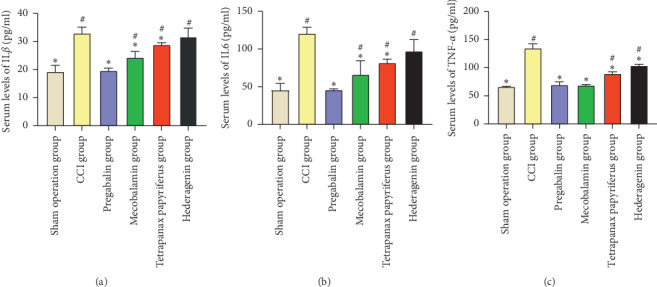
Serum IL6, IL1*β*, and TNF-*α* levels. ^#^The contrast of the sham operation group and the other groups. ^*∗*^Comparison of the CCI group and the other groups.

We detected serum IL6, IL1*β*, and TNF-*α* levels in all rats (Figure 5). In total, compared with the inflammation levels in the CCI group, three drugs, namely, pregabalin, mecobalamin, and *Tetrapanax papyriferus*, can reduce the levels of these three types of inflammatory factors in serum (Figures 5(a)–5(c), *p* < 0.05). Pregabalin was able to reduce their levels to normal, which was nearly the same level as the sham group, and the levels of IL1*β* and IL6 in other groups were different from those in the sham group (Figures 5(a) and 5(b), *p* < 0.05). After detecting the level of TNF-*α*, we found that pregabalin and mecobalamin could reduce its level to normal. Interestingly, hederagenin could reduce the level of TNF-*α* compared to this of CCI group (Figure 5(c), *p* < 0.05).

Pregabalin, mecobalamin, and *Tetrapanax papyriferus* can reduce the levels of IL6, IL1*β*, and TNF-*α* levels in serum (Figures 5(a)–5(c), *p* < 0.05). Compared with the levels of IL6 and IL1*β* in the sham operation group, pregabalin was able to reduce their levels to normal (Figures 5(a) and 5(b)), and the levels of IL1*β* and IL6 in other groups were different from those in the sham group. Also, pregabalin and mecobalamin could reduce its level to normal. Interestingly, hederagenin could reduce the level of TNF-*α* compared to those of the CCI group (Figure 5(c), *p* < 0.05).

### 3.7. The Expression of TRPA1, TRPM8, p38 MAPK, and Phosphorylated p38 MAPK

**Figure 6:**
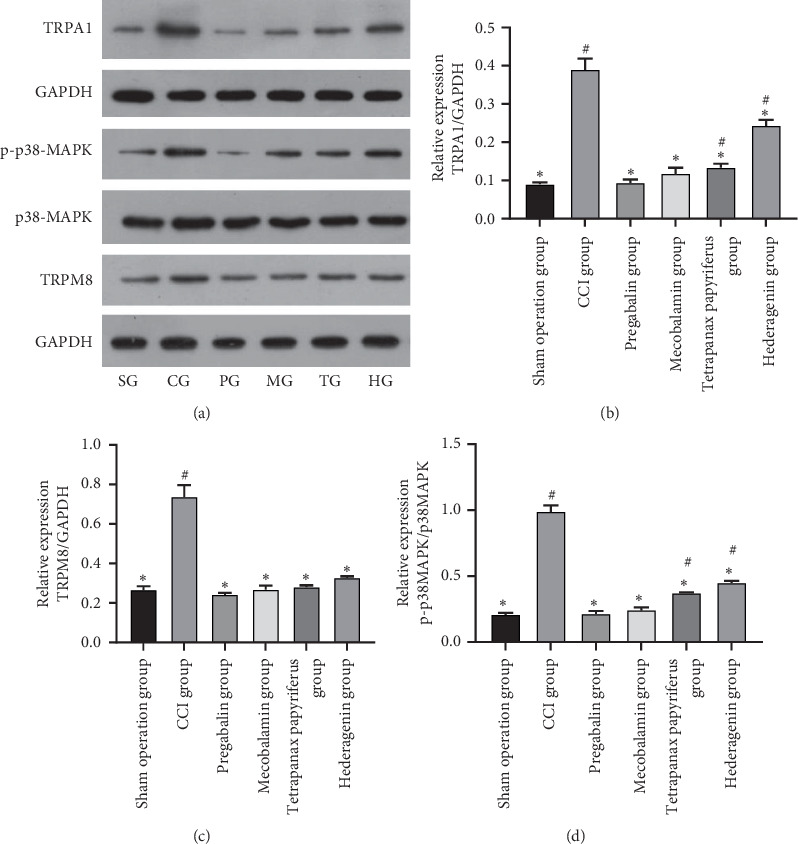
The expression of TRPA1, TRPM8, p38 MAPK, and phosphorylated p38 MAPK. ^#^The contrast of the sham operation group and the other groups. ^*∗*^Comparison of the CCI group and the other groups.

The expression of TRPM8 and TRPA1 in DRG increased evidently on the 21st day after the operation in the CCI group (Figures 6(a) and 6(b), *p* < 0.05). All four drugs could reduce the expressions of TRPM8 and TRPA1 compared to those of the CCI group (Figures 6(a) and 6(b), *p* < 0.05). All four drugs can reduce the level of TRPM8 to nearly normal levels as the level of the sham group (Figure 6(b), *p* < 0.05). Pregabalin and mecobalamin could reduce the level of TRPA1 to the normal, and others could not (Figure 6(a), *p* < 0.05). The level of phosphorylated p38 MAPK in DRG increased evidently in the CCI group (Figure 6(c), *p* < 0.05). Only pregabalin and mecobalamin could reduce the level of this to the normal, and the others could not (Figure 6(c), *p* < 0.05).

The expression of TRPM8, TRPA1, and phosphorylated p38 MAPK in DRG increased evidently on the 21st day after the operation in the CCI group. All four drugs could reduce the expressions of these compared to those of the CCI group (Figures 6(a)–6(c), *p* < 0.05). Pregabalin and mecobalamin could return the level of TRPM8, TRPA1, and phosphorylated p38 MAPK to the normal, and only *Tetrapanax papyriferus* and hederagenin returned the level of TRPM8 to the normal.

### 3.8. The Expression of TRPV1 and TRPV4

**Figure 7:**
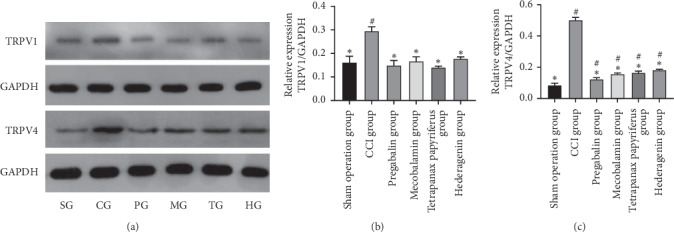
The expression of TRPV1 and TRPV4. ^#^The contrast of the sham operation group and the other groups. ^*∗*^Comparison of the CCI group and the other groups.

The expression of TRPV1 and TRPV4 in DRG increased evidently on the 21st day after the operation in the CCI group (Figures 7(a) and 7(b), *p* < 0.05). All four drugs could decrease the level of TRPV1 to the normal. Although these drugs could reduce the expression of TRPV4, they did not allow them to reach a normal level (Figures 7(a) and 7(b), *p* < 0.05) compared to the sham operation group.

The expression of TRPV1 and TRPV4 in DRG increased evidently on the 21st day after the operation in the CCI group (Figures 7(a) and 7(b), *p* < 0.05). Pregabalin and mecobalamin could decrease the levels of TRPV1 and TRPV4 to normal, and *Tetrapanax papyriferus* and hederagenin could do so (Figure 7).

## 4. Discussion

Chronic neuropathic pain is a serious and refractory disease, but the mechanisms involved remain obscure. Firstly, we performed KEGG enrichment analysis on chronic neuropathic pain-related proteins and got the conclusion that “inflammatory mediator regulation of TRP channels” might be most relevant to chronic neuralgia. The CCI model is a rat model of neuropathic pain based on a unilateral loose ligation of the sciatic nerve [20]. In experimental verification, we demonstrated that rats in CCI groups were more sensitive to hyperalgesia and allodynia to mechanical and thermal stimuli, as well as cold pain. In total, all four drugs, namely, pregabalin, mecobalamin, *Tetrapanax papyriferus*, and hederagenin, could relieve these pains. Compared with the pain sensitivity of the sham operation group, rats in pregabalin group on the 21st day after operation only appeared normal in hot plate experiment. By HE staining, we could hardly observe Schwann cells and see inflammatory cell infiltration normal in the normal structure of the sciatic nerve. However, the inflammatory cell infiltration was observed in the CCI model group, and proliferation of a large number of Schwann cells was also observed. Moreover, the normal structure of sciatic nerve was damaged. After the treatments, we observed a decrease in inflammatory cells and Schwann cell proliferation, and the morphology of the sciatic nerve tissue structure did not return to normal. Moreover, pregabalin, mecobalamin, and *Tetrapanax papyriferus* can reduce the levels of IL1*β*, IL6, and TNF-*α* in serum compared to those of CCI group. Besides, hederagenin could reduce the level of TNF-*α* compared to those of the CCI group. Pregabalin was able to reduce their levels to normal, but the levels of IL1*β* and IL6 in the other groups were different from those in the sham group. Also, pregabalin and mecobalamin returned the level of TNF-*α* to normal. The expression of TRPM8, TRPA1, TRPV1, TRPV4, and phosphorylated p38 MAPK in DRG increased evidently on the 21st day after the operation in the CCI group. All four drugs could reduce the expressions of these compared to those of the CCI group. Pregabalin and mecobalamin could reduce the level of TRPA1, TRPV1, TRPV4, and phosphorylated p38 MAPK to normal. Besides, *Tetrapanax papyriferus* and hederagenin returned the levels of TRPV1 and TRPM8 to normal. Levels of TRPA1 and phosphorylated p38 MAPK in *Tetrapanax papyriferus* and hederagenin groups were higher than those in the sham operation group. Also, these drugs could not make the expression of TRPM8 normal.

These drugs reduced expressions of TRP pathway proteins, including TRPA1, TRPM8, TRPV1, and TRPV4. TRPM8 and TRPA1 channel can be activated by cold temperatures or by a cooling agent [21, 22]. TRPA1 both as a cold receptor stimulus and as a sensitizer of mechanoreceptors was involved in the pathogenesis of neuralgia mechanical hyperalgesia. TRPM8 is required for the detection of cool to noxious cold temperatures as well as the expression of injury-induced cold allodynia and cooling-induced analgesia [4]. Besides, TRPM8 channels also play vital roles in the sensitization of nociceptive transduction [23]; they can amplify the pain signal. TRPV1 and TRPV4 are activated by distinct heat temperatures [9]. TRPV1 and TPRV4 are activated by mechanical stimuli as well and are, therefore, closely associated with mechanical hyperalgesia and thermal hyperalgesia [24].

Previous research has found that relieving neuropathic pain was regulated by inducing the release of inflammatory factors produced during central and peripheral inflammatory processes [11]. Inflammation is clearly the common denominator of pain, initiating the activation of several pathways that can trigger the transition from acute to chronic pain [25]. IL1*β*, IL6, and TNF-*α* could activate the TRP channel. Besides, TRPA1 and TRPV1 could increase the promotion of the secretion of IL1*β*, IL6, and TNF-*α* [26]. Both actions are achieved by promoting p38 phosphorylation. In this experiment, we observed differences in phosphorylation levels of p38 MAPK. Besides, levels of IL1*β*, IL6, and TNF-*α* in serum are consistent with the expression of the phosphorylation levels of p38 MAPK. Moreover, we also indicated that TRPs; levels of IL1*β*, IL6, and TNF-*α*; and phosphorylation of p38 MAPK have the same expression trends.

Chronic neuropathic pain resulting from peripheral nerve injury is characterized by pathological symptoms, such as hyperalgesia and allodynia to mechanical and thermal stimuli, as well as cold pain. We observed that these four drugs can alleviate these pains. And we can see that these drugs could reduce serum inflammatory factors, reduced the levels of elevated TRP proteins, and inhibit p38 MAPK phosphorylation, which may be related to the relief of neuralgia in CCI rats.

## 5. Conclusion

Rats in the CCI group showed hyperalgesia and allodynia to mechanical and thermal stimuli, as well as cold pain. And sciatic nerve structure was damaged. Besides, we observed high expressions of IL1*β*, IL6, and TNF-*α* in serum; high levels of TRPA1, TRPM8, TRPV1, TRPV4; and the phosphorylation of p38 MAPK. After treatment for about 21 days, these elevated expressions were reduced significantly, except the behavioral test results. We concluded that *Tetrapanax papyriferus* and hederagenin relieved sciatica by reducing inflammation levels, inhibiting p38 MAPK phosphorylation, and decreasing the levels of TRP proteins.

## Figures and Tables

**Table 1:** KEGG pathway analysis of chronic neuropathic pain-rated genes.

Gene set	Description	Size	Expected	Ratio
Hsa04750	Inflammatory mediator regulation of TRP channels	99	7.8336	7.5317
Hsa04912	GnRH signaling pathway	93	7.3588	6.6587
Hsa04720	Long-term potentiation	67	5.3015	6.4133
Hsa04540	Gap junction	88	6.9632	6.0317
Hsa04713	Circadian entrainment	96	7.5962	5.7924
Hsa01522	Endocrine resistance	98	7.7545	5.4162
Hsa04724	Glutamatergic synapse	114	9.0205	5.3212
Hsa04261	Adrenergic signaling in cardiomyocytes	144	11.394	5.0903
Hsa04725	Cholinergic synapse	112	8.8622	5.0777
Hsa04072	Phospholipase *D* signaling pathway	146	11.553	4.8474
Hsa04024	cAMP signaling pathway	199	15.746	4.8265
Hsa04722	Neurotrophin signaling pathway	119	9.4161	4.779

## Data Availability

The behavioral tests' data used to support the findings of this study are included within the supplementary information file.

## Abbreviations

TRP: Transient receptor potential
TRPV1: Transient receptor potential vanilloid 1
TRPV4: Transient receptor potential vanilloid 4
TRPM8: Transient receptor potential melastatin 8
TRPA1: Transient receptor potential ankyrin 1
CCI: Chronic constriction injury of sciatic nerve
DRG: Dorsal root ganglion
MWT: Mechanical withdrawal threshold
PWL: Paw withdrawal latency
HE: Hematoxylin-eosin
SG: Sham operation group
CG: CCI group
PG: Pregabalin group
MG: Mecobalamin group
TG: *Tetrapanax papyriferus* group
HG: Hederagenin group.

## Supplementary Materials

Supplementary Materials These data are about behavioral tests, which are related to Figure 3. Tables 1–3 are related to Figures 3(a)–3(c), respectively. These three tables and data can be drawn into those images.
