# Supplementary material for: Therapeutic Effect of Tetrapanax papyriferus and Hederagenin on Chronic Neuropathic Pain of Chronic Constriction Injury of Sciatic Nerve Rats Based on KEGG Pathway Prediction and Experimental Verification
Source: Evid Based Complement Alternat Med. 2020 Jun 13;2020:2545806. doi: 10.1155/2020/2545806 (PMC7306840; doi:10.1155/2020/2545806)
Supplement: Supplementary Materials — These data are about behavioral tests, which are related to Figure 3. Tables 1–3 are related to Figures 3(a)–3(c), respectively. These three tables and data can be drawn into those images. [file 2545806.f1.pdf]

Experimental supplement material

Table 1 Comparison of cold allodynia behavior in different groups of rats at different time points (times/minute)

	Group	-1d	1d	4d	7d	14d	21d
	SG	0.76±0.32	1.33±0.27	1.24±0.25*	0.86±0.26*	1.00 ±0.27*	0.71±0.56*
Acetone score	CG	0.67±0.43	1.19±0.18	5.24±0.66#	9.10±0.37#	11.29±1.26#	9.88±0.63#
	PG	0.67±0.34	1.38±0.23	2.38±0.23*	5.48±0.61*#	5.71±0.76*#	5.86±0.51*#
	MG	0.76±0.45	1.43±0.25	2.05±0.45*	6.24±0.90*#	6.00±0.67*#	6.52±0.74*#
	TG	0.72±0.12	1.43±0.25	2.19±0.42*	6.90±0.60*#	7.19±0.69*#	7.10±0.37*#
	HG	0.61±0.41	1.38±0.23	4.76±0.60*#	7.33±0.43*#	7.38±0.45*#	7.14±0.42*#

* states the contrast of CG and other groups, $p<0.05$. #means the comparison of SG and other groups，$p<0.05$.

Table 2 Comparison of mechanical allodynia behavior of rats in different groups at different time points

	Group	-1d	1d	4d	7d	14d	21d
	SG	23.29±0.69	16.46±1.28	23.02±0.51*	23.21±0.52*	23.49±0.54*	24.02±0.87*
50% MWT	CG	25.02±1.21	15.567±0.83	12.96±1.035#	7.62±0.92#	4.37±0.10#	3.96±0.56#
	PG	24.21±1.70	17.60±1.03	12.25±2.148#	11.70±0.44*#	9.62±0.90*#	14.80±0.76*#
	MG	23.59±1.83	16.98±1.60	11.00±1.60#	10.45±1.03*#	7.97±0.67*#	12.33±0.54*#
	TG	24.65±1.78	16.38±1.16	11.36±2.19#	10.95±1.28*#	7.55±0.60*#	8.98±0.44*#
	HG	24.21±1.82	16.00±1.32	11.91±1.82#	10.96±0.97*#	7.08±0.13*#	7.13±0.54*#

* states the contrast of CG and other groups，$p<0.05$. #means the comparison of SG and other groups，$p<0.05$. MWT: Mechanical withdrawal threshold

Table 3 Comparison of thermopathic behavioral behavior of rats in different groups at different time points (sec)

	Group	-1d	1d	4d	7d	14d	21d
	SG	19.09±0.78	15.19±1.52	17.91±0.70*	17.94±1.49*	18.00±1.06*	18.94±1.25*
	CG	19.61±0.63	14.42±1.89	6.81±0.46#	5.13±0.99#	4.61±0.76#	7.25±0.43#
PWL	PG	19.32±0.72	13.96±2.00	6.88±0.29#	10.07±1.18*#	13.69±1.459*	17.11±1.16*
	MG	19.58±0.60	14.10±2.72	7.00±0.80#	9.11±1.41*#	10.45±1.06*#	15.80±0.81*#
	TG	19.94±0.23	15.52±1.61	6.84±0.74#	7.23±0.62*#	8.81±0.98*#	14.14±0.42*#
	HG	19.78±0.71	14.05±1.60	6.86±0.72#	7.33±0.65*#	7.47±0.70*#	10.48±0.47*#

* states the contrast of CG and other groups，$p<0.05$. #means the comparison of SG and other groups，$p<0.05$. PWL：Paw withdrawal latency
